# Do guidelines on first impression make sense? Implementation of a chest pain guideline in primary care: a systematic evaluation of acceptance and feasibility

**DOI:** 10.1186/1471-2296-12-128

**Published:** 2011-11-21

**Authors:** Lena Kramer, Nagela Rabanizada, Jörg Haasenritter, Stefan Bösner, Erika Baum, Norbert Donner-Banzhoff

**Affiliations:** 1 Department of General Practice, Philipps University of Marburg, Germany

## Abstract

**Background:**

Most guidelines concentrate on investigations, treatment, and monitoring instead of patient history and clinical examination. We developed a guideline that dealt with the different aetiologies of chest pain by emphasizing the patient's history and physical signs. The objective of this study was to evaluate the guideline's acceptance and feasibility in the context of a practice test.

**Methods:**

The evaluation study was nested in a diagnostic cross-sectional study with 56 General Practitioners (GPs) and 862 consecutively recruited patients with chest pain. The evaluation of the guideline was conducted in a mixed method design on a sub-sample of 17 GPs and 282 patients. Physicians' evaluation of the guideline was assessed via standardized questionnaires and case record forms. Additionally, practice nursing staff and selected patients were asked for their evaluation of specific guideline modules. Quantitative data was analyzed descriptively for frequencies, means, and standard deviations. In addition, two focus groups with a total of 10 GPs were held to gain further insights in the guideline implementation process. The data analysis and interpretation followed the standards of the qualitative content analysis.

**Results:**

The overall evaluation of the GPs participating in the evaluation study regarding the recommendations made in the chest pain guideline was positive. A total of 14 GPs were convinced that there was a need for this kind of guideline and perceived the guideline recommendations as useful. While the long version was partially criticized for a perceived lack of clarity, the short version of the chest pain guideline and the heart score were especially appreciated by the GPs. However, change of clinical behaviour as consequence of the guideline was inconsistent. While on a concrete patient related level, GPs indicated to have behaved as the guideline recommended, the feedback on a more general level was heterogeneous. Several suggestions to improve guideline implementation were made by participating physicians. Due to the small number of practice nursing staff evaluating the flowchart and patients remembering the patient leaflet, no valid results regarding the flowchart and patient leaflet modules could be reported.

**Conclusions:**

Overall, the participating GPs perceived the guideline recommendations as useful to increase awareness and to reflect on diagnostic issues. Although behaviour change in consequence of the guideline was not reported on a general level, guidelines on history taking and the clinical examination may serve an important conservative and practical function in a technology driven environment. Further research to increase the implementation success of the guideline should be undertaken.

## Background

Chest pain is a frequent reason for consultation in primary care. The lifetime prevalence accounts for 20-40% of the general population [1] with an incidence of 0.7% in primary care [2]. Aetiologies for chest pain vary, with musculoskeletal, psychogenic, respiratory, and gastrointestinal causes being the most common underlying aetiologies. For most patients with chest pain, the general practitioner (GP) is the first person contacted in the health care system. Thus, the GP is confronted with the dilemma of identifying serious cardiac disease reliably while also fulfilling a gatekeeper role by protecting patients from unnecessary investigations, hospital admissions, and possible somatisation. The patient's medical history and the physical examination are of specific value for diagnosis, whereas the ECG and troponin test have a lower diagnostic benefit in primary care [3,4].

Guidelines for coronary heart disease (CHD) give specific recommendations. These often include investigations such as imaging and coronary angiography. However, the first step of the diagnostic process, the assessment of patient's medical history, is often neglected. Therefore, the Department of General Practice at the Philipps University of Marburg, Germany developed a guideline with emphasis on the patient's history and physical signs to explicitly address the diagnostic process in patients presenting with chest pain in primary care. The development process complied with the standards of the German Consortium of Scientific Medical Associations (AWMF) and the German College of General Practitioners and Family Physicians (DEGAM) [5,6]. Among other recommendations, the guideline included the Marburg Heart Score (see Table 1) to estimate the risk of a coronary heart disease [7].

**Table 1:** Components of the Marburg Heart Score

Score component	Assigned points
Age/gender (female ≥ 65, male ≥ 55)	**1**
Known clinical vascular disease	**1**
Patient assumes cardiac origin of pain	**1**
Pain worse with exercise	**1**
Pain not reproducible by palpation	**1**

1 point is assigned for each score variable. 3 different risk categories are derived:

low risk = 0-2 points; intermediate risk = 3 points; high risk 4-5 points.

In compliance with the DEGAM recommendations the guideline was developed stepwise, including a systematic literature review, the comments of experienced GPs, and a formal consensus with other specialty associations. A "practice test" to evaluate the guideline's acceptance and feasibility is also a relevant part of the guideline development programme. Therefore, the implementation experiences of GPs, practice nursing staff and patients should be assessed. This is of special interest as numerous studies have shown that guidelines and their implementation often fail to live up to expectations [8-12].

In our study ("practice test"), we were interested in the evaluation of the guideline's acceptance and feasibility referring to content and design, the issues influencing the guideline implementation, and potential behaviour change as a result of the guideline.

## Methods

### Description of the guideline

The chest pain guideline included a long version, a short version (two-page flowchart), a patient leaflet, and a flowchart for practice nursing staff. The patient leaflet was intended for patients with intermediate probability for coronary heart disease and is considered part of patient empowerment [13,14]. The flowchart gave the practice nursing staff instructions to triage patients regarding their need for emergency measures.

### Study design

Our study was embedded in a diagnostic cross-sectional study to validate a heart score with 56 GPs and 862 consecutively recruited patients with chest pain (publication in progress). In compliance with the DEGAM recommendations regarding the guideline development we conducted a sub study ("practice test") to evaluate the guideline's acceptance and usability by GPs, practice nursing staff, and patients. This evaluation study lasted 12 weeks per GP and was undertaken between October 2009 and February 2010. In this context, the participating GPs were invited to take part in two focus groups held in January and February 2010. Both focus groups (FG1 and FG2) were moderated by an external GP and one of the authors, neither of which was involved in the development of the chest pain guideline. Prior to the evaluation study, the participating GPs were invited to a seminar and received the guideline material. To obtain a case-related assessment of the benefits and appropriateness of the guideline, GPs were instructed to list every patient presenting with chest pain in a case record form that included three questions regarding the application of the guideline. GPs and patients were informed in detail about the study and both gave their written consent to the study participation. At the end of the recruiting period, GPs and nursing staff were asked to answer standardized questionnaires assessing their overall evaluation of the guideline modules. GPs reviewed the long and short version; practice nursing staff reviewed the flowchart. Patients who received a patient leaflet were asked for their evaluation of the leaflet via phone interview. Ethical approval for the study was obtained from the Ethics Committee of the Faculty of Medicine of Philipps University of Marburg, Germany.

### Participants and recruitment procedures

**Figure 1:**
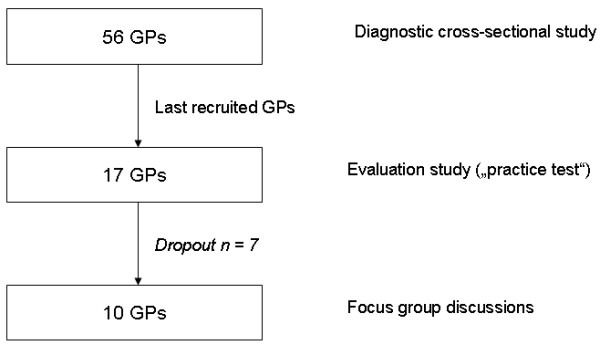
**Flow trial of the GP sample**.

Due to the explorative character and practical considerations as the guideline was not yet consented at the beginning of the cross-sectional study we asked the 17 last recruited GPs (all asked GPs gave their consent to the study participation) of the 56 GPs in the cross-sectional study to participate in our evaluation study. We considered one third of the original sample as appropriate for our sub-study as there were no requirements in the DEGAM recommendation about the sample size of the practice test. The flow trial of the sample is shown in Figure 1.

All physicians were located in the Marburg region of Germany and were recruited from the regional physician network of the Philipps University of Marburg's Department of General Practice. To assure representiveness, we insured that an appropriate mix of participating GPs, e.g., rural vs. urban region and female vs. male, took part in the study.

### Data collection

According to the taxonomy of mixed methods as outlined by Palinkas [15], we sequentially collected quantitative (standardized questionnaires, case record forms) and qualitative data (focus group discussions). We used qualitative interviews to answer questions raised by quantitative data (function: expansion). In a final step, we linked the information gained from our qualitative data with the quantitative data set (process: connect).

The standardized questionnaires covered main issues of the guideline and had to be answered on a 7 step Likert scale from 1 = "not at all" to 7 = "very much", or with yes or no (dichotomous) indicating the agreement with the statement (see Tables 2 and 3). This feedback informed the following focus groups, where further insights into the guideline implementation process were to be gained [16].

**Table 2:** Results of the GP questionnaire - 7 step Likert scale (n = 17)

Question	1	2	3	4	5	6	7	Median	SD*
	"not at all"						"very much"		
To what extent did you become familiar with the guideline content?	-	-	2	-	11	1	3	5	1.13
How much do you agree with the main recommendations of the guideline?	-	-	-	-	8	2	7	6	.97
How do you evaluate the clarity of the guideline's long version?	-	1	5	-	8	-	3	5	1.54
How do you evaluate the practical relevance of the guideline's long version?	-	-	2	2	7	-	6	5	1.41
How do you evaluate the comprehensibility of the guideline's long version?	-	-	1	2	5	2	7	6	1.31
How do you evaluate the suitability of the guideline's long version for the general practice?	1	-	2	-	10	-	4	5	1.58
How do you evaluate the clarity of the guideline's short version?	-	-	1	-	3	4	9	7	1.13
How do you evaluate the practical relevance of the guideline's short version?	-	-	1	-	2	4	10	7	1.11
How do you evaluate the comprehensibility of the guideline's short version?	-	-	1	-	3	3	10	7	1.15

* = Standard deviation

**Table 3:** Results of the GP questionnaire - dichotomous (n = 17)

Question	no	yes
Is there a need for a chest pain guideline?	3	14
Is the targeted patient group clearly defined?	1	16
Illnesses that are rare in general practice are neglected in the guideline. Does that make sense?^+^	-	16
Do you think this guideline is interesting because it contains new aspects for you?	4	13
Do you think this guideline is convenient as a memory aid?	3	14
Do you think this guideline is dispensable because you have always acted accordingly?	14	3
Do you think this guideline is dispensable because it's not realizable?	16	1

^+ ^= one value is missing

The focus group discussions were based on a semi-structured guideline developed jointly by two of the authors and based on previously conducted studies by our department on changes of professional behaviour [17,18] and the results of the GPs' questionnaire (Tables 2 and 3). Main topics of the focus groups discussion guideline were the evaluation of the chest pain guideline and its key recommendations, suggestions for improvement, key factors that influence the implementation of the chest pain guideline in a negative or positive way, and intended behaviour changes as a result of the chest pain guideline.

Focus group participants were assured that their responses would remain confidential and anonymous. The process of planning and conducting the qualitative analysis followed the recommendations as outlined by Kuckartz et al. [19].

The standardized approach in quantitative and qualitative data collection and moderation of both focus groups by the same external GP and one of the authors contributed to minimizing bias in data collection.

### Data analysis

The questionnaires for GPs and practice nursing staff, the answers for patients' phone interviews to evaluate the patient leaflet, and the guideline-related case record form items were analyzed descriptively for frequencies, means, and standard deviations. Calculations were conducted via SPSS and Microsoft Excel.

Focus group discussions were audio-taped and transcribed verbatim. The accuracy of transcripts was checked prior to being transferred to the computer software Maxqda 2007, which assisted data handling [20]. Based on the discussion guideline, a thematic coding frame for the data analysis was developed, repeatedly checked and, if necessary, supplemented by additional categories that emerged during the analyzing process [21]. Transcripts were coded separately by two of the authors and then checked for consistency. The data analysis and interpretation followed the standards of the qualitative content analysis [22,23]. The translation of questionnaires and focus group discussions form German to English was conducted by a native English speaker.

## Results

### Study population

The majority of the 17 GPs participating in the study were male (77%), between 41 and 50 years of age (59%), and in practice full-time (71%). The subgroup of 10 focus group participants, of which 3 participated in FG1 and 7 in FG2, showed a similar demographic distribution. We did not identify the reasons for non-participation of 7 GPs. Further characteristics of the participants are summarized in Table 4.

**Table 4:** GP study population (evaluation study: n = 17, focus groups: n = 10)

	Evaluation study		Focus groups	
Demographics and professional characteristics	*n*	***(%)***^***a***^	*n*	***(%)***^***a***^
**Gender**				
Male	13	(77)	8	(80)
Female	4	(24)	2	(20)

**Age (years)**				
41 to 50	10	(59)	5	(56)
51 to 60	5	(29)	3	(33)
> 60	1	(6)	1	(11)

**Established for (years)**				
≤ 10	7	(41)	5	(50)
11 to 20	5	(29)	3	(30)
> 21	3	(18)	2	(20)

**Characteristic of the practice**				
Single practice	4	(24)	1	(10)
Group practice	12	(71)	9	(90)

**Practice location**				
< 5000	3	(18)	2	(20)
5000 to 20,000	6	(35)	2	(20)
20,000 to 100,000	7	(41)	6	(60)

**Status**				
Full time	12	(71)	6	(66)
Part time	4	(24)	3	(33)

^a ^Numbers may not add up to 17 and percentages may not add up to 100% due to missing values and rounding.

Overall, 282 patients with chest pain were recruited by the participating practices. A total of 15 patients (11 female, 4 male) refused to participate. With 136 women and 131 men, the gender ratio was nearly balanced. The average age amounted to 58.2 years (standard deviation: 13.3).

### Results of GPs questionnaire, case record form items and focus groups

As the study was undertaken to gain insights into the guideline's acceptance and feasibility, we focused on the identification of general factors by using cross-case analysis and were less interested in interindividual differences. Table 1 illustrates frequencies, means, and standard deviations of the GPs' questionnaire items, referring to the evaluation of the guideline's short and long version.

#### Content and design of the chest pain guideline

According to the GPs' questionnaire, the agreement with the guideline recommendations was high or very high for all participating GPs. Fourteen participants were convinced that there was a need for this kind of guideline and perceived guideline recommendations as useful. While the short version of the guideline was evaluated as good or very good (regarding clarity, practical relevance, and comprehensibility), the feedback for the long version was more heterogeneous. Six GPs criticized the long version as lacking in clarity. Practical relevance and comprehensibility of the long version were perceived positively by 13 and 14 participants, respectively.

In line with the reported results of the questionnaire, the short version of the guideline and the heart score were appreciated by the focus group participants.

*"I found the short version very good and very clearly presented. I also like the layout and [its use] for structuring the diagnostic process so that you don't leave out anything" *[FG2]

*"As a general direction, as a table, as an algorithm to follow, it [the heart score] is a nice, supportive tool." *[FG2]

*"It [short version] is actually a small guideline in itself, helpful to quickly orient [oneself] because it's not as complicated as the big guideline... I found it simple, yet still reliable." *[FG1]

*"But these five points [of the heart score] really impressed me. And I found them valid in most cases." *[FG1]

Concerning the guideline's long version, the feedback of the participants was partially more critical. While the content-related completeness of the long version was well received, its layout and clearness were criticized.

*"The older you get, the more work experience you have, the more structured you work. And this is represented in this guideline quite simply and without leaving out anything." *[FG1]

*"I laid it [the long version] aside after two thirds [of the text]; never looked at it again. I found it really terrible." *[FG2]

Individual GPs criticized that the troponin test wasn't generally recommended as a useful diagnostic tool. Guideline authors refrained to include the troponin test due to insufficient sensitivity within the first hours of acute coronary syndrome. Physicians also missed suggestions for adequate handling with co-morbidity (e.g., chest pain and mental problems) and risk factors (e.g., diabetes, smoking).

*"I think the troponin test is missing there." *[FG2]

*"[...] if mental problems coincide, than it becomes really difficult. [...] and there the guideline is too imprecise" *[FG2]

*"[...] if someone is diabetic and is over fifty, then he has a higher baseline risk; especially if he is a smoker." *[FG2]

#### Implementation issues and change of behaviour

As shown in the GP's questionnaire, the suitability of the chest pain guideline for general practice was evaluated positively by 14 GPs. Three participants felt there was no added value by the guideline because they would already behave according to guideline recommendations.

**Figure 2:**
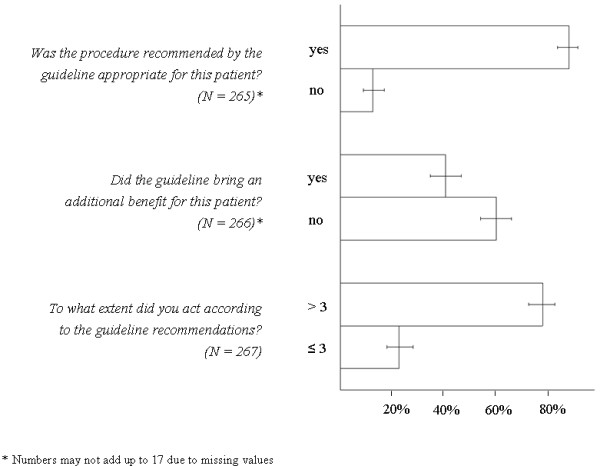
**Results of the guideline related case record form items**.

Results of the three case record form items related to the chest pain guideline, analyzed for 267 patients, are illustrated in Figure 2.

In the majority of cases, GPs perceived guideline recommendations to be appropriate. An additional benefit by the guideline was seen for 107 patients. GPs mentioned multimorbid patients among the reasons for the lack of an additional value. Overall, the participating physicians assumed to have managed actual patients according to the guideline recommendations.

When asked for their behaviour change as a consequence of studying the guideline on a more general level, the participants of the focus groups gave heterogeneous feedback. While some GPs reported being more conscious and attentive in the consultation, others didn't recognize a behaviour change because they typically would have treated their patients according to guideline recommendations.

*"The whole thing [the guideline] led to a more intensive examination of the clinical picture, especially with the medical history or the things you have just found out." *[FG2]

*"What was written in those new guidelines is actually what I've done all my life." *[FG2]

To improve implementation chances of the chest pain guideline, GPs proposed developing a PC version of the heart score or offering regular trainings and a desk version of the heart score.

*"[...] I think it would be good to establish a culture of permanent professional development or training." *[FG1]

"It [PC-version] would just remind one to bring the heart score to mind."

The balance between "cookbook medicine" - marked by a high adherence to the guideline's recommendations - and individual treatment of the patient was an essential point for the interviewed physicians. Even though participants appreciated the value of standardised guidelines, they rated the consideration of intuition and experience in the consultation as very important.

*"Like all standards, it reminds one to not overlooking something important. When I think how our work could become better, then I think we should internalize check lists, like a pilot. I think this would be progress in our field." *[FG2]

"*On the other hand, I am not a fan of cookbook medicine-ticking off things. That's not my type of general practice. A principle problem of general practice is dealing with many individual patients; how you can balance it." *[FG1]

*"It's important to be aware of your behaviour and check where you might oversee something or where routine might have crept in. To this extent, a process of awareness and reflection is good." *[FG2]

The flowchart evaluation questionnaire for practice nursing staff was answered by only a small number of participants so that no valid results can be reported. Phone interviews with the 27 patients who received a patient leaflet were not analyzable because a majority didn't remember the patient leaflet.

## Discussion

Based on questionnaires, case record forms, and focus group discussions, the overall evaluation of the GPs participating in the evaluation study regarding the recommendations made in the chest pain guideline, was positive. While the long version was partially criticized for a perceived lack of clarity, the short version and the heart score were especially appreciated. Reported change of behaviour as consequence of the guideline was inconsistent. Several suggestions to improve guideline implementation were made.

A remarkable aspect of the guideline evaluation concerns the heterogeneous feedback of the long version. As the focus group discussions revealed, some GPs criticized the layout and perceived lack of clarity of the long version, whereas they did not generally refuse the recommendations. Nevertheless, individual GPs were reluctant to accept some recommendations, despite the fact that its content (e.g., regarding the troponin test) is based on solid evidence derived from several studies. A reason may be the complex presentation since the diagnostic effectiveness of each item from the history must be discussed in relation to several outcomes. Another reason may be that GPs are used to randomized controlled trials (RCT) informing therapeutic decisions, but not a patient's history and physical signs; these are still regarded as areas for intuition.

Due to low levels of feedback from nurses and patients regarding the flowchart and patient leaflets, respectively, the benefit of these modules must be questioned. A reason for the low acceptance of the flowchart may be found in the low collaboration of nursing staff and GPs in Germany. Most German practices are small, with one to three GPs; therefore, nursing staff typically have little scope for decisions so that a patient presenting with chest pain is immediately referred to a GP. Another reason could be that an informal rule how to treat patients with chest pain is already implemented within the practices so that the flowchart seems to have no additional benefit for the nursing staff.

GPs' reasons for their low use of the patient leaflet needs to be investigated in further research and, if necessary, the leaflet's content and/or layout should be modified. Since patient interviews were performed six weeks after the index-consultation, the few patients who received a leaflet may have forgotten its use and content. Whether the patient leaflet reduced patients' anxiety in our research, like a study of Arnold and colleagues [24] showed in the setting of a hospital emergency department, can not be definitely answered. It is possible that chest pain patients presenting to their GP are less anxious than patients presenting to the emergency department. As Jones and Mountain recommend, further research regarding the benefits of patients leaflet should be undertaken [13].

According to the findings of other authors [8-10], a high quality of guidelines and the agreement of GPs are no guarantors for a successful implementation of guidelines. The heterogeneous feedback concerning the perceived additional diagnostic value by the guideline and the physicians' behaviour change in consequence of the guideline knowledge reveals that agreement alone is not a sufficient precondition for a lasting implementation of the chest pain guideline [10,12,25].

The attitude of the physician towards guidelines plays an important role in the decision to implement guideline recommendations. This assumption is in line with the theory of planned behaviour, where attitude, in addition to subjective norm and perceived behavioural control, is an important predictor for behavioural intention [26]. A variety of studies have shown the theory's relevance for the medical sector [27-29].

Some of the participating GPs reported that they don't recognize noticeable differences between the guideline recommendations and their own previous behaviour. Thus, to increase implementation success of guidelines, significant diagnostic or treatment innovations should be indicated by a well-arranged design of the guideline (e.g., desk version of the heart score), so that differences to previous behaviour become obvious. A reason for the different feedback concerning GPs behaviour change on a concrete (see case record forms (CRFs)) and more general level (see questionnaires and focus groups) might be that the CRFs were filled in directly after the consultation, so the specific behaviour was more present to the GPs, while the general feedback, in retrospect, was more prone to recall bias.

Being aware that the GPs' perception of conformity doesn't necessarily correspond to real facts, further research must be undertaken to investigate a supposed perception-reality gap. Additional recommendations on how to improve the design and evaluation of medical innovations are proposed by Murray and colleagues in their normalisation process theory (NPT) [30]. NPT shall serve as a sensitizing tool, enabling researchers to think through issues of implementation while designing and evaluating complex interventions. By integrating interventions into routine work implementation potential is enhanced.

However, as opposed to further laboratory investigations, behaviour regarding first clinical assessment of a patient is difficult to define. Questions and expert reasoning help specify the probabilities for relevant conditions. Deviations from the standard proposed by the guideline cannot be observed within a study design of this kind, since most reasoning occurs inside GPs' thought process.

### Study limitations

Our study was not based on a conceptual framework. Nevertheless, due to the research question and the evaluative and pragmatic character of the study, we presume the chosen method to be appropriate.

As a result of the small and non-randomized sample, the representativeness of the data may be limited. The low participation rate of GPs in the focus group discussions (10 out of 17 GPs) was most likely due to the additional effort required to visit our department after a long work day.

Social desirability may have biased focus group discussions and answer patterns from the questionnaires causing the reported evaluation of the guideline to perhaps be rated more positively than it actually was. Another limitation concerns the lacking psychometric evaluation of the used questionnaires, although we carefully considered standard format. For confidentiality reasons we could not match focus group contributions with the questionnaire answer pattern of the participating GPs.

## Conclusions

The overall evaluation of the reported chest pain guideline was positive. Participating GPs perceived the guideline as a welcome opportunity to increase awareness and encourage reflection of diagnostic issues. We assessed different data on physician and patient levels in the evaluation; reasons for criticism on single modules were discussed. In consequence of our study, portions of the chest pain guideline were modified corresponding to the participating GPs' feedback.

By focussing on the assessment of symptoms and the patient's medical history as first steps in the diagnostic process, our guideline focuses on a core domain of general practice: giving GPs confidence regarding their clinical skills and routines in an area with high pressure to refer and to employ technology.

## Competing interests

The authors declare that they have no competing interests.

## Authors' contributions

All authors contributed to study design. JH, SB and NDB developed the chest pain guideline. LK and NDB drafted the discussion guideline for the focus groups. LK moderated the focus group discussions. Data coding and analysis of the qualitative data were coordinated and performed by LK and NR. Quantitative data analysis was performed by LK and JH. LK drafted the manuscript. JH, SB, NR, NDB and EB provided critical review on all parts of the manuscript. All authors read and approved the final version of the manuscript.

## Pre-publication history

The pre-publication history for this paper can be accessed here:

http://www.biomedcentral.com/1471-2296/12/128/prepub
